# Direct estimation of central aortic pressure from measured or quantified mean and diastolic brachial blood pressure: agreement with invasive records

**DOI:** 10.3389/fcvm.2023.1207069

**Published:** 2023-07-25

**Authors:** Daniel Bia, Federico Salazar, Luis Cinca, Marcos Gutierrez, Alvaro Facta, Yanina Zócalo, Alejandro Diaz

**Affiliations:** ^1^ Departamento de Fisiología, Centro Universitario de Investigación, Innovación y Diagnóstico Arterial (CUiiDARTE), Facultad de Medicina, Universidad de la República, Montevideo, Uruguay; ^2^ Sección Hipertensión Arterial, Departamento de Cardiología, Hospital Privado de Comunidad, Mar del Plata, Argentina; ^3^ Instituto de Investigación en Ciencias de la Salud, UNICEN-CCT CONICET, Tandil, Argentina

**Keywords:** aortic pressure, brachial blood pressure, catheterism, invasive records, non-invasive records, oscillometry, physiological measurements, human physiology

## Abstract

**Background:**

Recently it has been proposed a new approach to estimate aortic systolic blood pressure (aoSBP) without the need for specific devices, operator-dependent techniques and/or complex wave propagation models/algorithms. The approach proposes aoSBP can be quantified from brachial diastolic and mean blood pressure (bDBP, bMBP) as: aoSBP = bMBP^2^/bDBP. It remains to be assessed to what extent the method and/or equation used to obtain the bMBP levels considered in aoSBP calculation may affect the estimated aoSBP, and consequently the agreement with aoSBP invasively recorded.

**Methods:**

Brachial and aortic pressure were simultaneously obtained invasively (catheterization) and non-invasively (brachial oscillometry) in 89 subjects. aoSBP was quantified in seven different ways, using measured (oscillometry-derived) and calculated (six equations) mean blood pressure (MBP) levels. The agreement between invasive and estimated aoSBP was analyzed (Concordance correlation coefficient; Bland-Altman Test).

**Conclusions:**

The ability of the equation “aoSBP = MBP^2^/DBP” to (accurately) estimate (error <5 mmHg) invasive aoSBP depends on the method and equation considered to determine bMBP, and on the aoSBP levels (proportional error). Oscillometric bMBP and/or approaches that consider adjustments for heart rate or a form factor ∼40% (instead of the usual 33%) would be the best way to obtain the bMBP levels to be used to calculate aoSBP.

## 1. Introduction

Several approaches and devices are used to non-invasively estimate aortic systolic blood pressure (aoSBP). They differ in the technology (e.g., ultrasound, applanation tonometry), recording-site (e.g., carotid, radial, brachial) and/or in the mathematical analysis (e.g., direct vs. general transfer function-derived estimation) considered (1–4). This could result in differences in the aoSBP levels obtained (1, 2) and in the agreement with invasive aoSBP data. This, in turn, could depend on the “calibration scheme” considered (1, 2, 5–9). However, at present there is no consensus and it is still discussed on which (if any) would be the best approach to estimate aoSBP (1, 2). The above could have contributed to the fact that aoSBP estimation has not become widespread in clinical practice, despite the recognized value of knowing central haemodynamics in different situations (10).

Recently, Chemla et al. proposed a simple approach [“direct central blood pressure estimation (DCBP)”] that could “facilitate” aoSBP estimation (and may help to expand its use in clinical practice). According to the authors, from brachial diastolic (bDBP) and mean (bMBP) blood pressure (BP), aoSBP could be determined as: aoSBP = bMBP^2^/bDBP (11). It should be noted that prior to becoming “accepted and generalized”, the proposed method must be further evaluated by contrasting the aoSBP values it estimates with those obtained invasively (catheterization). On the other hand, and related with the above, it should be evaluated to what extent the way in which bMBP is quantified could impact on the aoSBP levels estimated. In this regard, mean blood pressure (MBP), could correspond to MBP (or bMBP) *measured* by oscillometry (lowest cuff pressure value measured during the maximum oscillations' plateau), or *calculated* from bDBP and bSBP (1, 2, 12). About this, bMBP has been calculated from equations that differ in the use of predefined empirical adjustments (e.g., adding 5 mmHg to pre-calculated values), corrections by heart rate (HR) and/or in the form factor (FF) considered (e.g., 33%, 42%) (12–14). bMBP levels obtained with different approaches can differ significantly and it should be noted that while some authors have stated that a given approach would be superior to the others when estimating bMBP, others suggested that ´the best´ way to quantify bMBP may differ depending on the situation and/or the aim pursued (12, 15–17).

The objective of the present study was to assess the level of association and agreement between aoSBP obtained invasively (catheterization) and estimated (DCBP), considering different approaches to quantify bMBP. It is worth noting that in this work we are not validating a calculated parameter, but providing information on what margins of deviation and error could be expected if the aortic pressure quantification approach (“DCBP”) proposed by Chemla et al. is used in clinical settings.

## 2. Methods

### 2.1. Subjects

Eighty-nine subjects undergoing coordinated coronary angiogram (Department of Cardiology of the Hospital Privado de Comunidad, Mar del Plata, Argentina) were included. Aortic valve disease, left ventricular (LV) outflow tract obstruction and/or arrhythmia were exclusion criteria. Prior to the study, a clinical evaluation enabled assessing the exposure to cardiovascular risk factors (18–23). All the included subjects gave their written informed consent. Data included in this work were not considered in prior publications. The protocol was approved by the Institutional Ethic Committee. The procedures agreed with the Declaration of Helsinki.

The following data were obtained: (1) invasive aortic BP (aoBP) and bBP (catheterization), (2) non-invasive bBP and aoBP, levels and waveforms assessed from oscillometric/plethysmographic brachial artery data (Mobil-O-Graph device, Model PWA, IEM GmbH, Stolberg, Germany).

### 2.2. Invasive measurement of aoBP and bBP

Intra-arterial aoBP and bBP levels and waveforms were obtained with the subjects lying in supine position. Asepsis of the area, followed by cutaneous/subcutaneous injection of lidocaine was performed prior to the arterial (radial) access. Then, a 5 or 6 French introducer sheath was positioned in the arterial lumen and heparin was administered. After that, a 0.035-inch guide wire was placed in the ascending aorta and finally a 5 French pig tail catheter (Cordis, Miami, USA) was introduced. The catheter tip was always placed ∼4 cm away from the aortic valve. Once the correct positioning of the catheter was verified (fluoroscopy), the guide was removed and the catheter was washed with saline solution. Soft sedation was administered during the catheterization to minimize pain and discomfort.

To obtain intravascular (proximal ascending aorta or brachial) pressure, the fluid-filled catheter was connected to an external transducer (TruWave, PX260, Edwards, Dominican Republic), associated to a Mindray Mec 2012 system (Shenzhen Mindray Bio-Medical Electronics Co., China) which was synchronized with a x-ray device (Allura CV-20, Philips Healthcare, Netherlands). The external transducer was always kept at the heart level (mid-axillary line) and was calibrated in agreement with the system's inbuilt two-point calibration technique. The Allura CV-20 monitor allowed a display of the registered BP waves. Prior to any record or measurement the system was flushed with saline solution and the quality of the pressure signals was visually checked.

After obtaining aoBP data, the catheter was placed in the brachial artery (opposite to that of the limb of the vascular access), at the level in which the cuff for bBP measurement was positioned. Then, invasive intra-arterial bBP was measured and non-invasive (oscillometry-derived) bBP values were obtained immediately before or after the invasive recordings. After each bBP recording, the catheter was placed in the ascending aorta to check hemodynamic stability.

From invasive BP data, the processing systems enabled HR, systolic and diastolic BP values to be obtained.

After data collection, the catheter was withdrawn and the patient was sent to the recovery area.

### 2.3. Non-invasive measurement of bBP and MBP estimation

Immediately before and/or after each invasive aortic or brachial record, bBP was non-invasively determined from a pneumatic cuff positioned in the arm opposite to that of the vascular access (oscillometry/plethysmography, Mobil-O-Graph device) (20, 24, 25). The system obtains bMBP (and HR) and after applying internal algorithms (manufactureŕs property) it gives systolic (bSBP) and bDBP (but not the bMBP), from which pulse pressure can be calculated (bPP, bPP = bSBP-bDBP).

From the data obtained, bMBP was quantified as follows (1, 15, 26, 27):

(i) bMBP_0.42 _= 0.42*bSBPosc + 0.58*bDBPosc
(ii) bMBP_0.412 _= bDBPosc + [0.412*(bSBPosc-bDBPosc)]
(iii) bMBP_0.33 _= bDBPosc + 0.33*(bSBPosc-bDBPosc).
(iv) bMBP_0.33 + 5 _= bDBPosc + [0.33*(bSBPosc-bDBPosc) + 5].
(v) bMBP_0.33HR _= bDBPosc + [0.33 + (0.0012*HRosc)]*(bSBPosc-bDBPosc)
(vi) bMBP_SBP*DBP_^0.5 ^= (bSBPosc*bDBPosc)^0.5^

The suffix *osc* was used to name the variables obtained from oscillometry (bMBPosc, bSBPosc, bDBPosc, bPPosc and HRosc). The bMBP values obtained as described were considered to calculate the aoSBP using Chemla et al. approach (DCBP) (11).

### 2.4. Estimation of aoSBP

Using the equation proposed by Chemla et al. (11), and considering the different methods used to calculate MBP, aoSBP levels were obtained from invasive aoBP, invasive bBP and non-invasive bBP recordings. The non-invasive bBP data used to calculate aoBP were obtained simultaneously with the invasive aortic recordings. Then, as an example, aoSBP obtained from non-invasive bBP was named according to the approach used to quantify bMBP: (i) aoSBP_0.42, (ii) aoSBP_0.412, (iii) aoSBP_0.33, (iv) aoSBP_033 + 5, (v) aoSBP_0.33HR, (vi) aoSBP_SBP*DBP^0.5^ and (vii) aoSBP_Osc_._

### 2.5. Data analysis

#### 2.5.1. Association and agreement between measured and estimated aoSBP

After analyzing the subjectś characteristics (Table 1; Supplementary File S1, Supplementary Table S1), we evaluated the association and agreement between aoSBP data invasively measured and estimated. To this end, Lin's Concordance Correlation Coefficient (CCC) and Bland-Altman analyses (Table 2; Supplementary File S1, Supplementary Tables S2–S4; Supplementary File S2, Supplementary Figures S1–S3) were considered. Measured (invasive) aoSBP was always the ´reference method´. The analyses were performed for aoSBP levels (DCBP), calculated from systolic and diastolic BP values: (i) measured at the aorta (Supplementary Figure S1), (ii) measured at the brachial artery (Supplementary Figure S2), and (iii) estimated from non-invasive bBP records (Mobil-O-Graph) (Supplementary Figure S3). Bland-Altman tests were used to assess the presence of mean (systematic) and proportional errors between aoSBP data obtained with the reference (invasive) and the ´tested´ method (DCBP). The analyses correspond to reference data (measured aoSBP; x-axis) plotted against the difference between measured and estimated aoSBP (y-axis). The regression equations were obtained.

**Table 1:** Demographic, anthropometric and clinical characteristics of the study population.

Variable	MV	ME	SD	Min	p25th	p50th	p75th	Max
Sex (Female, %)	36.8%							
Age	66	1	10	37	60	67	73	85
Body height (cm)	168	1	9	145	160	168	175	190
Body weight (Kg)	81	1	16	52	70	77	92	123
BMI (Kg/m^2^)	28.62	0.37	4.52	21.64	25.00	28.09	31.35	42.06
Glicemia	110	2	28	81	95	103	116	256
Total cholesterol (mg/dl)	180	5	49	105	143	176	206	353
LDL cholestrol (mg/dl)	103	4	45	26	71	96	122	278
HDL cholesterol (mg/dl)	50	1	12	27	41	49	56	84
Triglycerides (mg/dl)	163	10	101	63	107	142	191	766
Creatinine (mg/dl)	0.94	0.02	0.21	0.57	0.78	0.91	1.04	1.48
Blood urea nitrogen (mg/dl)	39	2	15	21	29	36	46	100
Cardiovascular Major Event (%)	50.0							
Peripheral arterial disease (%)	13.2							
Hypertension (%)	82.9							
Dyslipidemia (%)	75.0							
Smoke, Never (%)	57.9							
Smoke, Current (%)	17.1							
Smoke, Ex (%)	25.0							
Diabetes (%)	23.7							
Sedentarism (%)	93.4							
Obesity (%)	34.2							
Familiy History CVD (%)	4.0							
Drugs, Hypertension (%)	85.5							
Drugs, Dyslipemia (%)	65.8							
Drugs, Diabetes (%)	21.1							
Invasive aortic and brachial blood pressure: arterial-catheterization								
aoSBP_inv (mmHg)	135	2	26	88	116	135	151	220
aoDBP_inv (mmHg)	70	1	12	44	61	70	77	107
aoPP_inv (mmHg)	65	2	21	15	51	63	79	128
aoHR_inv (beats/minute)	65	1	10	44	58	64	71	95
bSBP_inv (mmHg)	135	2	24	92	117	132	152	189
bDBP_inv (mmHg)	68	1	11	43	60	69	75	107
bPP_inv (mmHg)	67	2	20	17	52	67	80	118
bHR (beats/minute)	64	1	9	44	58	63	69	92
Non-invasive brachial blood pressure: oscillometry								
bSBP (mmHg)	137	2	21	101	118	135	152	188
bDBP (mmHg)	80	1	13	49	70	80	89	111
bPP (mmHg)	57	1	16	25	44	55	66	101
bHR (beats/minute)	64	1	10	44	56	64	71	96

MV, mean value; SD, standard deviation; Min and Max, minimum and maximum value; SD, standard deviation; p25th, p50th and p75th, percentiles 25, 50 (median) and 75, respectively; BMI, body mass index; CVD, cardiovascular disease; SBP, DBP, PP and MBP, systolic, diastolic, pulse and mean blood pressure, respectively; HR, heart rate; Inv, invasive derived; suffix: ao, aorta; b, brachial artery.

**Table 2:** Correlation and agreement between invasive aoSBP (reference) and estimated aoSBP (considering invasive aoBP and invasive and non-invasive bBP levels, and different bMBP equations).

(1) Agreement between invasive aoSBP and aoSBP obtained using invasive aoBP and different bMBP equations							
Method	aoSBP_AO_Inv_033 (mmHg)		aoSBP_AO_Inv_033 + 5 (mmHg)	aoSBP_AO_Inv_033HR (mmHg)	aoSBP_AO_Inv_042 (mmHg)	aoSBP_AO_Inv_0412 (mmHg)	aoSBP_AO_Inv_(sbp*dbp)^0.5^ (mmHg)
Concordance correlation							
CCC	0.819		0.993	0.987	0.982	0.988	1.000
Pearson	0.999		0.998	0.989	0.989	0.991	1.000
Pearson, *p* value	<1.0E-14		<1.0E-14	<1.0E-14	<1.0E-14	<1.0E-14	0.00
Bland-Altman (Reference—Method)							
ME (mmHg)	14.20		0.55	0.79	−1.85	−0.33	0.00
ME, *p* value	<1.0E-14		0.0494	0.0492	9.75E-05	0.42	1.00
Regression Eq.	−1.40 + 0.11x		−12.12 + 0.09x	6.54–0.04x	10.08–0.08x	8.96–0.06x	——-
Slope, *p* value	<1.0E-14		<1.0E-14	1.13E-02	1.83E-06	3.35E-05	——-
(2) Agreement between invasive aoSBP and aoSBP obtained using invasive bBP and different bMBP equations							
Method	aoSBP_BA_Inv_033 (mmHg)		aoSBP_BA_Inv_033 + 5 (mmHg)	aoSBP_BA_Inv_033HR (mmHg)	aoSBP_BA_Inv_042 (mmHg)	aoSBP_BA_Inv_0412 (mmHg)	aoSBP_BA_Inv_(sbp*dbp)^0.5^ (mmHg)
Concordance correlation							
CCC	0.82		0.93	0.93	0.91	0.92	0.94
Pearson	0.95		0.94	0.93	0.93	0.94	0.95
Pearson, *p* value	<1.0E-14		<1.0E-14	<1.0E-14	<1.0E-14	<1.0E-14	<1.0E-14
Bland-Altman (Reference—Method)							
ME (mmHg)	11.54		−2.30	−2.65	−5.54	−3.92	−3.03
ME, *p* value	<1.0E-14		7.30E-03	4.60E-03	7.57E-08	4.80E-05	2.79E-04
Regression Eq.	−14.46 + 0.19x		−25.77 + 0.17x	−8.67 + 0.04x	−6.29 + 0.01x	−7.09 + 0.02x	−15.97 + 0.09x
Slope, *p* value	6.22E-09		2.71E-07	0.2578	0.8916	0.5532	4.70E-03
(3) Agreement between invasive aoSBP and aoSBP obtained using non-invasive bBP and different bMBP equations							
Method	aoSBP_033 (mmHg)	aoSBP_033 + 5 (mmHg)	aoSBP_033HR (mmHg)	aoSBP_042 (mmHg)	aoSBP_0412 (mmHg)	aoSBP_(sbp*dbp)^0.5^ (mmHg)	aoSBP_Osc (mmHg)
Concordance correlation							
CCC	0.64	0.79	0.80	0.83	0.82	0.83	0.83
Pearson	0.86	0.86	0.85	0.86	0.86	0.86	0.86
Pearson, *p* value	<1.0E-14	<1.0E-14	<1.0E-14	<1.0E-14	<1.0E-14	<1.0E-14	<1.0E-14
Bland-Altman (Reference—Method)							
ME (mmHg)	13.14	0.55	3.01	1.15	2.28	−0.71	−4.30
ME, *p* value	<1.0E-14	0.684	0.027	0.369	0.078	0.583	0.001
Regression Eq.	−46.5 + 0.44x	−58.08 + 0.43x	−47.01 + 0.37x	−45.82 + 0.34x	−45.89 + 0.35x	−49.18 + 0.35x	−45.34 + 0.30x
Slope, *p* value	<1.0E-14	<1.0E-14	1.71E-13	3.51E-13	7.21E-14	7.48E-14	5.58E-10

ME, mean error; CCC, Lińs Concordance Correlation Coefficient. The Bland-Altman tests included (i) on the ordinates (y-axis) the difference between invasively measured (reference) and estimated (tested method) aoSBP (in the order: invasive minus non-invasive), and (ii) on the abscissa (x-axis) the value of aoSBP recorded invasively by catheterization (reference or gold standard technique). In the concordance correlation analyses, the invasively measured aoSBP was placed on the y-axis.

#### 2.5.2. Level of agreement between invasive and estimated aoSBP

The measurements were divided into four categories (ranges) according to Bland-Altman mean errors (rounded absolute value): (i) 0–5 mmHg, measurements considered “very accurate” (errors without clinical relevance; green), (ii) 6–10 mmHg, measurements “slightly inaccurate” (yellow), (iii) 11–15 mmHg, measurements “moderately inaccuraté (orange), and (iv) >15 mmHg, “very inaccurate” measurements (red) (Table 3) (11, 28). Taking into account these bands and the regression equation obtained in the Bland-Altman analysis, the bias (in mmHg) for each of the methods used to quantify aoSBP was determined considering the minimum, the maximum and the percentiles 25th, 50th and 75th of the invasive aoSBP data. (Table 3, Top). In addition, the ranges of measured aoSBP levels for which the different estimation methods would yield “very accurate” (−5 to 5 mmHg), “slightly inaccurate” (−10 to 10 mmHg) or “moderately inaccurate” (−15 to 15 mmHg) estimates were identified (Table 3, Bottom). Finally, the “average” aoSBP levels for which the different estimation methods would achieve the described errors were calculated.

**Table 3:** Non-invasive approach error levels related to invasive blood pressure values.

(1) Errors (invasive—calculated) for different aoSBP levels invasively obtained						
	Invasive aoSBP (see Table 2)					
	Min	p25th	p50th	p75th	Max	
Approach	88 mmHg	116 mmHg	135 mmHg	151 mmHg	220 mmHg	Equation (*)
aoSBP_033 (mmHg)	−8	5	13	20	51	y = −46.51 + 0.44x
aoSBP_033 + 5 (mmHg)	−20	−8	1	7	37	y = −58.08 + 0.43x
aoSBP_033HR (mmHg)	−14	−4	3	9	34	y = −47.01 + 0.37x
aoSBP_042 (mmHg)	−15	−5	1	7	31	y = −45.82 + 0.34x
aoSBP_0412 (mmHg)	−15	−5	2	8	33	y = −45.89 + 0.35x
aoSBP_(sbp*dbp)^0.5^ (mmHg)	−18	−8	−1	5	30	y = −49.18 + 0.35x
aoSBP_Osc (mmHg)	−19	−10	−4	1	22	y = −45.34 + 0.30x
(2) Ranges of invasive aoSBP for which non-invasive estimation ensure a very accurate (−5 to 5 mmHg), slightly inaccurate (−10 to 10 mmHg) or moderately inaccurate (−15 to 15 mmHg) measurement						
Associated error (mmHg)	−15	−10	−5	5	10	15
aoSBP_033 (mmHg)						
Invasive aoSBP (mmHg):	71	82	94	117	127	140
aoSBP_033 + 5 (mmHg)						
Invasive aoSBP (mmHg)	99	110	122	146	158	169
aoSBP_033HR (mmHg)						
Invasive aoSBP (mmHg)	86	99	113	141	155	168
aoSBP_042 (mmHg)						
Invasive aoSBP (mmHg)	88	102	117	147	161	176
aoSBP_0412 (mmHg)						
Invasive aoSBP (mmHg)	86	100	114	144	158	172
aoSBP_(sbp*dbp)^0.5^ (mmHg)						
Invasive aoSBP (mmHg)	94	108	122	152	166	180
aoSBP_Osc (mmHg)						
Invasive aoSBP (mmHg)	99	115	132	167	183	200
Average all methods (mmHg)						
Invasive aoSBP (mmHg)	89	102	116	145	158	172
Standard deviation	9.7	10.7	11.7	14.9	16.7	17.9

Top (1): In the regression equation, “y” indicates the error (difference) between measured and estimated aoSBP (mmHg), and “x” value represents the invasively recorded aoSBP value (mmHg). (*): equation resulting from the Bland-Altman analysis. The difference (error) was categorized into four bands according to its rounded absolute value: 0–5 mmHg (green: measurements considered “very accurate” or no error of clinical relevance); 6–10 mmHg (yellow: measurements considered “slightly inaccurate”); 11–15 mmHg (orange: measurements considered “moderately inaccurate”); >15 mmHg (red: measurements considered “very inaccurate”). Bottom (2): Associated error: the value indicates the error (invasive—estimated aoSBP; mmHg); the colour indicates the error category (band): 0–5 mmHg (green); 6–10 mmHg (yellow), 11–15 mmHg (orange).

Evans's Empirical Classification (“correlation strength”) was used to interpret r values: <0.20: very weak; 0.20–0.39: weak; 0.40–0.59: moderate; 0.60–0.79: strong; ≥0.80: very strong (29). According to the central limit theorem, taking into account Kurtosis and Skewness coefficients distribution and the number of subjects (sample size > 30) a normal distribution was considered (30). The sample size exceeds the minimum (*n* = 85) recommended for studies in which analyses of agreement between invasive and non-invasive BP measurements are performed (17). MedCalc (v.14.8.1, MedCalc Inc., Ostend, Belgium) and IBM-SPSS Statistical Software (v.26, SPSS Inc., Illinois, USA) were used. A *p* < 0.05 was considered as the statistical significance threshold.

## 3. Results

### 3.1. Population and hemodynamic characteristics

The studied subjects were distributed over a wide range of ages (37–85 y) (Table 1; Supplementary File S1, Supplementary Table S1). Invasive aoSBP and bSBP values were also distributed across a broad range: 7.8% and 4.5% were <100 mmHg, 53.9% and 48.4% were between 100 and 139 mmHg, 21.3% and 30.3% were between 140 and 159 mmHg, and 16.9% and 15.7% of the values were ≥160 mmHg. In turn, invasive aoDBP and bDBP values were <60 mmHg in 21.3% and 25.8% of cases; they were between 60 and 84 mmHg in 68.5% and 64.0%, and in 10.1% and 8.9% of cases the values were >85 mmHg. HR values were always within the expected (normal) range.

### 3.2. Agreement between measured and estimated aoSBP

#### 3.2.1. Aortic invasive records

When considering invasive aoDBP and aoMBP calculated from invasive aoSBP and aoDBP (Table 2; Supplementary File S1, Supplementary Table S2, Supplementary File S2, Supplementary Figure S1: (i) calculated and measured (invasive) aoSBP data showed ´very stronǵ association, (ii) the equation using a FF = 33% showed a large mean error (14.2 mmHg), whereas (iii) the equations using a FF = 33% with HR correction, a FF = 42% or a FF = 41.2% achieved very low mean errors (0.3 to 1.85 mmHg). The equation aoMBP = (aoSBP*aoDBP)^0.5^ allowed full agreement with invasive aoSBP. With the only exception of the latter, all the methods showed proportional errors. The largest was observed when using FF = 33%.

#### 3.2.2. Brachial invasive records

Similarly, when analyzing invasive bDBP and bMBP calculated from invasive bSBP and bDBP records (Table 2; Supplementary File S1, Supplementary Table S3, Supplementary File S2, Supplementary Figure S2): (i) calculated and invasive aoSBP showed “very strong” association, (ii) using a FF = 33% resulted in large mean error (11.5 mmHg), and (iii) equations using FF = 33% and HR correction, FFs = 42% and FF = 41.2%, achieved very low mean bias (<5 mmHg), and showed no proportional errors.

#### 3.2.3. Brachial non-invasive (oscillometric) records

Invasive and non-invasive aoSBP data showed “strong” and “very strong” degrees of concordance (CCC between 0.64 and 0.83). The lowest CCC (yet statistically significant) was observed when analyzing invasive aoSBP and DCBP quantified using bMBP_033 (FF = 33% without HR correction) (Table 2; Supplementary File S1, Supplementary Table S4, Supplementary File S2, Supplementary Figure S3).

The mean error values obtained with the different methods were distributed over a wide range (−4.30 to 13.14 mmHg). However, most (6 out of 7) approaches showed mean bias between −5 and 5 mmHg and several (4 out of 7) showed mean errors without statistical significance (Table 2). The highest mean error was observed for aoSBP quantified using bMBP_033 (FF = 33% uncorrected for HR).

The “slopes” (proportional errors) of the linear adjustments showed that regardless of the equation used to quantify bMBP, aoSBP data obtained from bMBP^2^/bDBP showed variations in the error levels related to inter-individual differences in invasive aoSBP (Table 2).

### 3.3. Agreement between measured and estimated aoSBP

Related with the above, when analyzing the errors observed when considering different aoSBP levels it was found that (Table 3, Top): (i) all approaches underestimated aoSBP (error range: 22–51 mmHg) at high aoSBP levels (e.g., close to 220 mmHg, the maximum measured). The highest bias was obtained when using FF = 33% without correction for HR; (ii) at low invasive aoSBP levels (e.g., close to 88 mmHg, the lowest value measured) all approaches overestimated aoSBP (error range: 8–20 mmHg); (iii) for aoSBP values within 25th and 75th percentiles (116–151 mmHg), non-invasive approaches allowed reaching errors <10 mmHg (except for the method using a FF = 33%). The calculation of aoSBP using bMBPosc would enable to minimize errors when considering high invasive aoSBP levels (Table 3, Top); but the use of bMBP calculated using FF = 33% corrected for HR, FF = 42% or 41.2%, would result in acceptable bias.

Table 3 (Bottom), shows that different non-invasive approaches had different aoSBP ranges in which they “ensured” errors between (i) −5 and 5 mmHg (green), (ii) −10 and 10 mmHg (yellow) and/or (iii) −15 and 15 mmHg (orange). For instance, estimating aoSBP from bDBP and bMBPosc, allowed ensuring errors <10 mmHg when invasive aoSBP levels were between 115 and 183 mmHg, while the calculus of bMBP using an FF = 33%, would ensure reduced errors within an aoSBP range between 82 and 127 mmHg. In general terms, the remaining methods (FF = 33% corrected for HR, FF = 42% or 41.2%) resulted in errors <10 mmHg, within a pressure range of 100–110 (lower limit) and 155–165 mmHg (upper limit). In summary, the different approaches used to calculate aoSBP: (i) showed differences in “global” mean bias, (ii) over- and under-estimated aoSBP at low and high BP levels, respectively, and (iii) showed differences in the aoSBP range in which they would perform best as aoSBP estimators.

## 4. Discussion

### 4.1. Clinical and physiological relevance

Chemla et al. developed their equation by comparing invasive (cathetersm) measurements in the ascending aorta with invasive measurements in the radial and brachial arteries. The validation of the Chemla et al. equation for cuff-based oscillometrically measured bBP values is still pending. Additionally, it remains to be assessed to what extent the method or equation used to obtain the bMBP levels has an influence on the validity of the Chemla et al. equation. In this work we applied, for the first time using invasive and non-invasive records, the method proposed by Chemla et al. and analyzed the obtained data with the aim of contributing to define to what extent the approach considered to determine the bMBP values to be used to calculate the aoSBP according to the method would impact on the accuracy and validity of the estimated data. The main contribution of this manuscript is the demonstration that the usefulness of the method recently proposed by Chemla et al. would be (i) highly dependent on the approach used to quantify bMBP, and (ii) on the aoBP levels considered. Our work highlights four issues.

First, the ability of Chemla et al. (11) approach to obtain aoSBP values close to those measured invasively depends on the way in which bMBP is obtained (measured or quantified) and on the actual aoSBP levels in the specific subject. Then, trying to generalize and define dichotomously whether the approach is “good or bad” without taking into account the above would be a mistake (and an over-simplification). The different approaches used to calculate aoSBP: (i) showed differences in “global” mean bias, (ii) over- and under-estimated aoSBP at low and high BP levels, respectively, and (iii) showed differences in the aoSBP range in which they would perform best as aoSBP estimators.

Second, calculating bMBP using a FF = 33% without HR adjustment (the most widespread way of calculating the MBP), would result in aoSBP values far from the invasive ones. Furthermore, that approach gave the highest mean error levels. Additionally, compared to other approaches, its best performance (lowest error) was observed at low aoSBP levels (Table 3, Bottom) which would be mainly observed in haemodynamic states or clinical situations in which assessing central haemodynamics could not be considered decisive (e.g., in terms of clinical decisions). On the other hand, and in the same line, at least in theory, aoSBP_033 could be considered useful to assess aoSBP in children and adolescents who have low aoSBP. However, it would not be useful in children/adolescents exposed to clinical conditions and/or risk factors (e.g., sedentary, overweight-obesity) in which aoSBP levels have been shown to be elevated (25, 31–33). This should be evaluated in future studies.

Third, the other methods used to calculate bMBP and/or bMBPosc, showed (quite) similarity in their ability to estimate aoSBP. Unfortunately, most brachial cuff-based methods (oscillometric devices) do not give bMBPosc, even though it is quantified as a prior step to the obtaining of bSBP and bDBP (the values actually given). In fact, most of the oscillometric devices do not show the researcher or clinician the bMBPosc (e.g., Mobil-O-Graph, Omron semi-automatic BP devices). These devices show on the display the HR and bSBP and bDBP values (calculated with the manufacturer's own internal algorithms). Then, the researcher and/or clinician can only quantify bMBP using equations such as those used in our manuscript. In other words, the systems “measure” the bMBPosc (as is widely known), but then use it to calculate bSBP and bDBP values, which are the values shown, and do not display the measured data. Therefore, the bMBPosc related approach while accurate would be difficult to apply and generalize in clinical practice.

Fourth, methods using a FF = 33% with HR correction and/or a FF close to 40% (42% or 41.2%) may be one step ahead of the rest when jointly considering three factors: (i) agreement with invasive aoSBP, (ii) aoSBP range within which they ensure the lowest errors (100–110 to 155–165 mmHg), and (iii) feasibility to be applied in clinical practice.

### 4.2. Strengths and limitations

First, healthy subjects were not included in this work. This is a common feature of this kind of studies given the conditions required for the indication of invasive evaluations (e.g., suspected or known cardiovascular disease) (2). However, and in line with the above, the studied subjects would be representative of those whose accurate hemodynamic and/or cardiovascular assessment would be considered critical in clinical practice.

Second, the sample size (*n* = 89) exceeded the minimum recommended for studies aimed at analyzing the agreement (e.g., Blant-Altman test) between invasive and non-invasive BP measurements (17). In addition, despite, the sample size could be considered moderate, it enabled to detect statistical differences, thus achieving suitable statistical power (minimizing type 2 errors). Measurements in the brachial artery opposite to that of the vascular access limb and the need for additional recordings in the aorta considered in the study protocol, increased catheterization-time, which restricted the number of patients considered elective and/or who agreed to participate.

Third, although we are aware that differences between measured and estimated aoSBP could vary depending on covariates (34, 35) neither the sample size, nor its heterogeneity allowed to define subgroups (e.g., defined by age, sex and/or exposure to risk factors) and perform adequate statistical analyses. Further multicentre studies would be necessary to analyze the impact of covariates on the results.

Fourth, we used “fluid column” transducers instead of solid-state pressure sensors, which characteristically provide accurate BP waveforms (mainly due to their ability to detect the high-frequency components of the wave). In any case, fluid column transducers are not only the sensors used in our Hospital but they are widely used to measure aoSBP in clinical practice. Furthermore, in the ARTERY Society task force consensus statement on protocol standardization, Sharman et al. stated that while micromanometer-tipped catheters would be the sensors of choice, if carefully handled, fluid column catheters could be used to measure intra-arterial BP (17). Additionally, recently, in a systematic review and meta-analysis, fluid-filled and catheter-tipped transducers have shown similar mean bias in non-invasive aoSBP estimation (2). Taking into account the natural frequency and damping coefficient of our recording (catheter-tubing-external transducer) system, and although the methods and devices used are widely validated, the systolic and diastolic BP values obtained invasively could entail a small over- and under-estimation, respectively.

Fifth, an issue to consider is that regardless of the method used, non-invasively assessed bBP always has “inherent errors” (e.g., under- and over-estimation of bSBP and bDBP, respectively) (36). Then, the ability of bBP to accurately quantify aoBP (using the method of Chemla et al.) may depend (among other factors) on the approach and device used. Additionally, taking into account the inter-individual differences in BP amplification and in the brachial pulse waveform, the form factor that should be used to properly calculate bMBP may vary (37). In this regard, Schultz et al. showed that no universal form factor would achieve an accurate estimation of bMBP in all individuals (37). Thus, our results regarding the best approach to quantify bMBP to be used to estimate aoSBP must be analyzed in the context of the overall scenario, as there may be differences among individuals.

## 5. Conclusions

The ability of “aoSBP = MBP^2^/DBP” equation to accurately estimate (error <5 mmHg) invasive aoSBP levels depends on the bMBP method/equation employed, and on the actual aoSBP levels (proportional error).

The best way to obtain bMBP to be used to calculate aoSBP would be bMBPosc and/or approaches that include adjustments for HR or FF ∼40% (bMBP_042_, bMBP_0.412_, bMBP_033HR_), instead of the usual FF = 33%.

## Data Availability

The raw data supporting the conclusions of this article will be made available by the authors, without undue reservation.
