# Supplementary material for: Direct estimation of central aortic pressure from measured or quantified mean and diastolic brachial blood pressure: agreement with invasive records
Source: Front Cardiovasc Med. 2023 Jul 25;10:1207069. doi: 10.3389/fcvm.2023.1207069 (PMC10409477; doi:10.3389/fcvm.2023.1207069)

**Figure S1.** Agreement between aoSBP levels (i) measured invasively (catheterization) and (ii) calculated (aoSBP=aoMBP^2^/aoDBP) from invasive measurements of aoSBP and aoDBP: concordance correlation plots (Top) and Bland-Altman plots (Bottom)

Concordance correlation coefficient plots


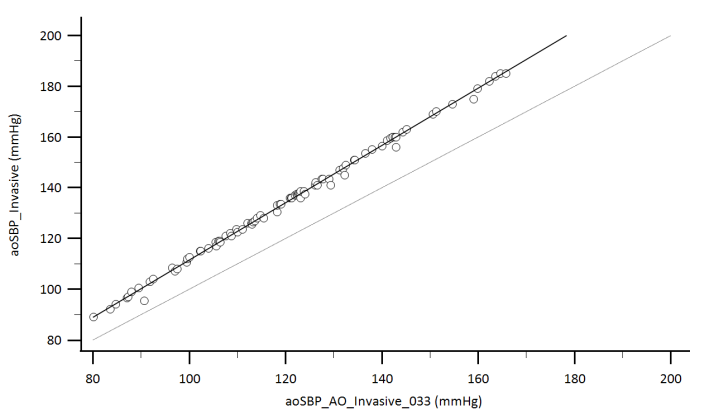

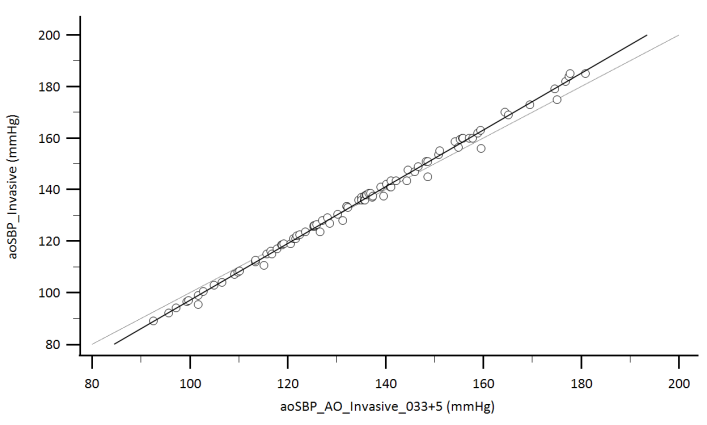


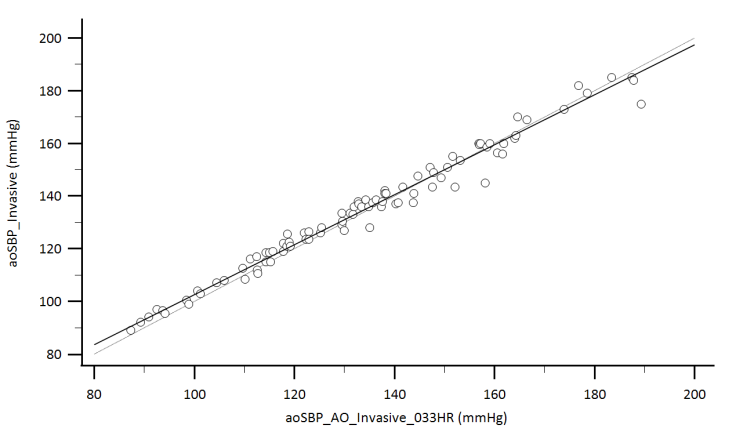

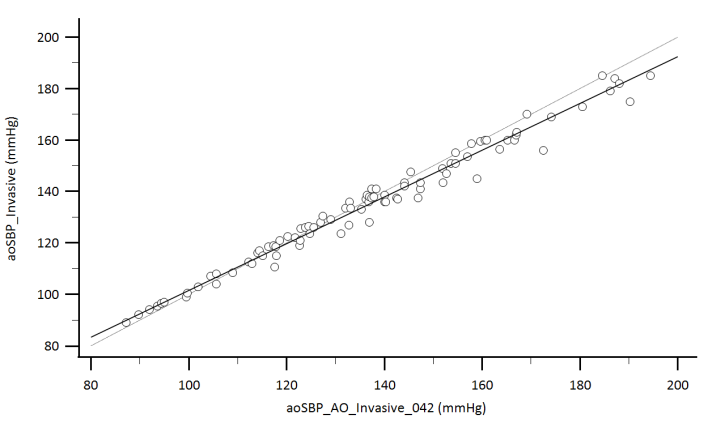


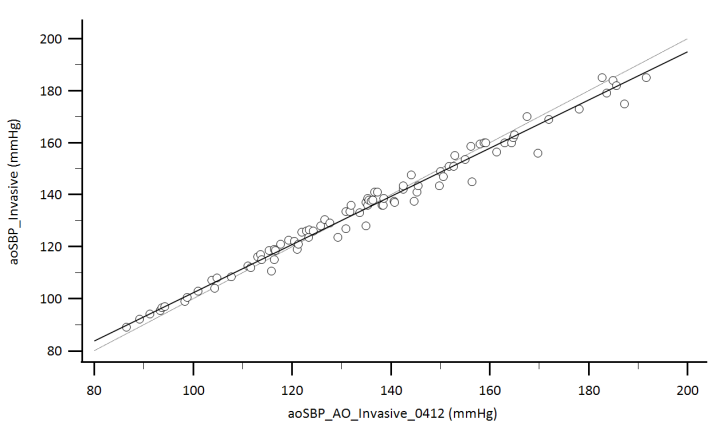

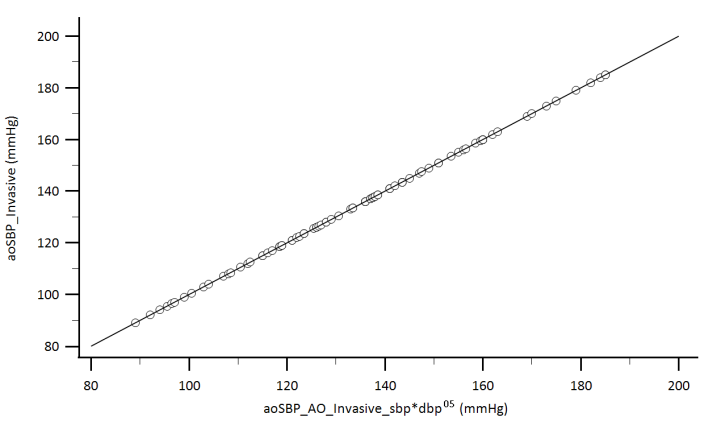


Bland Altman plots


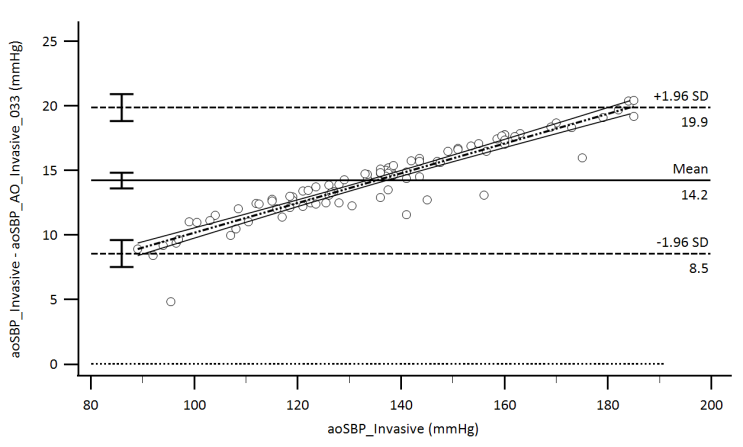

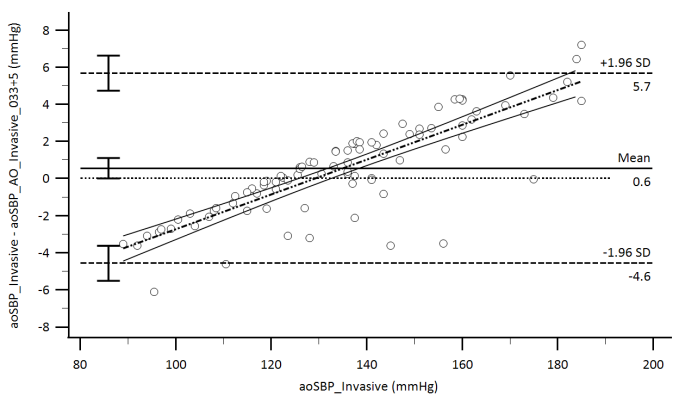


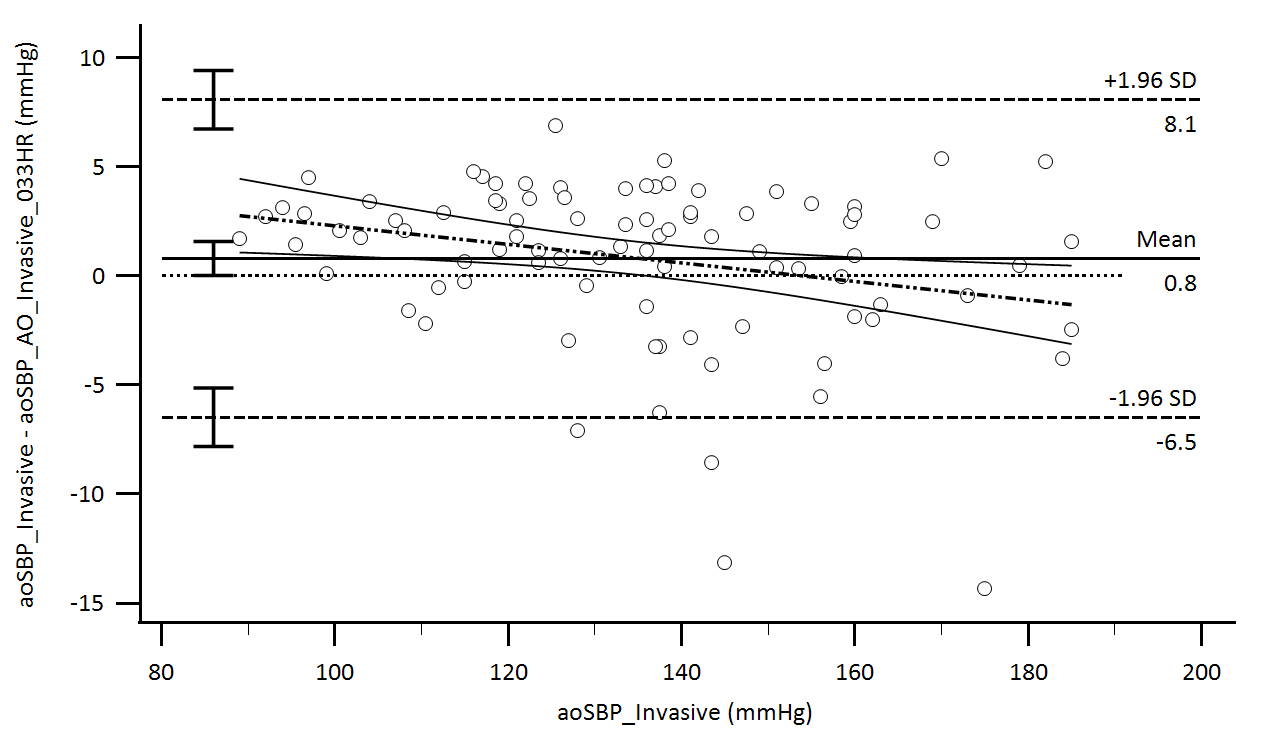

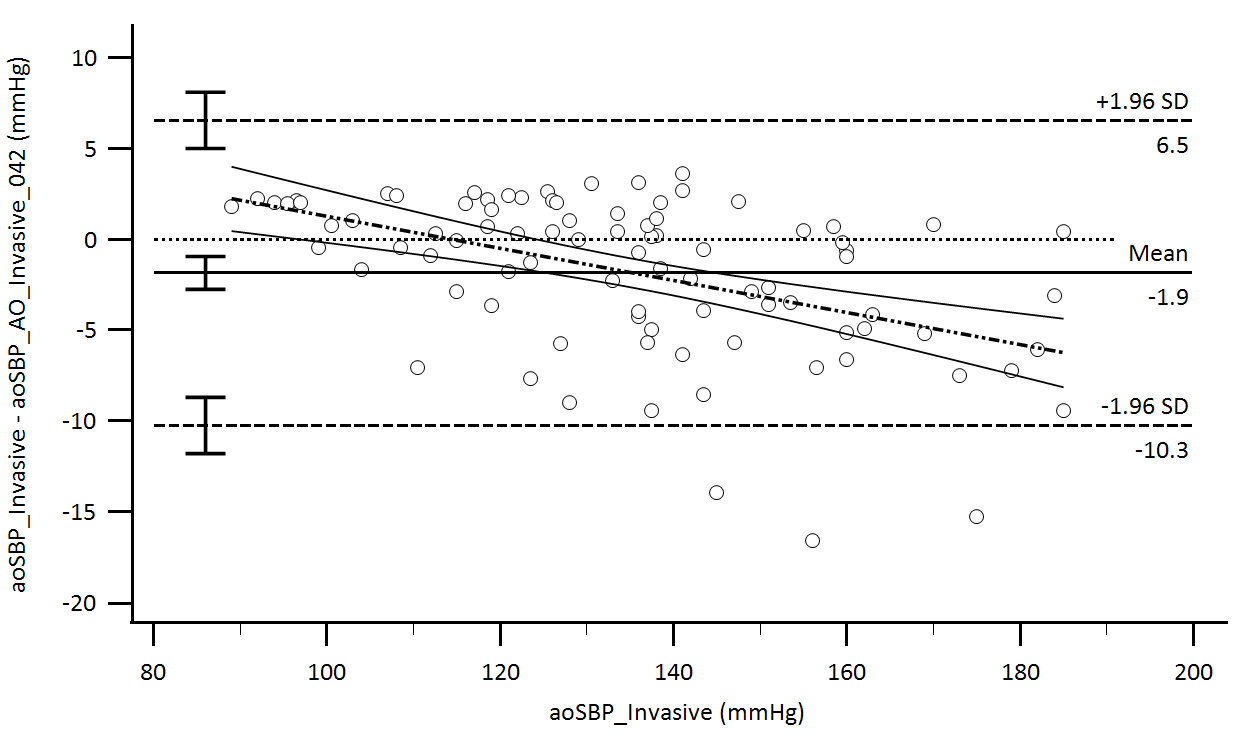


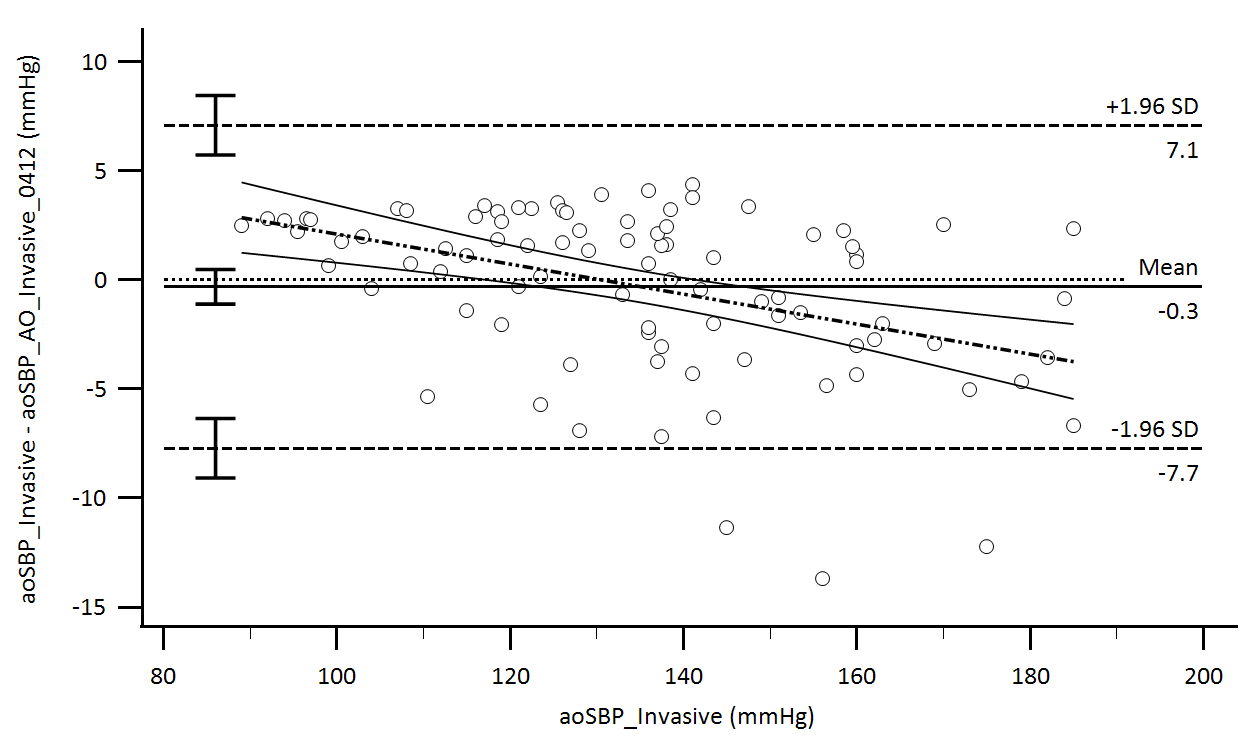

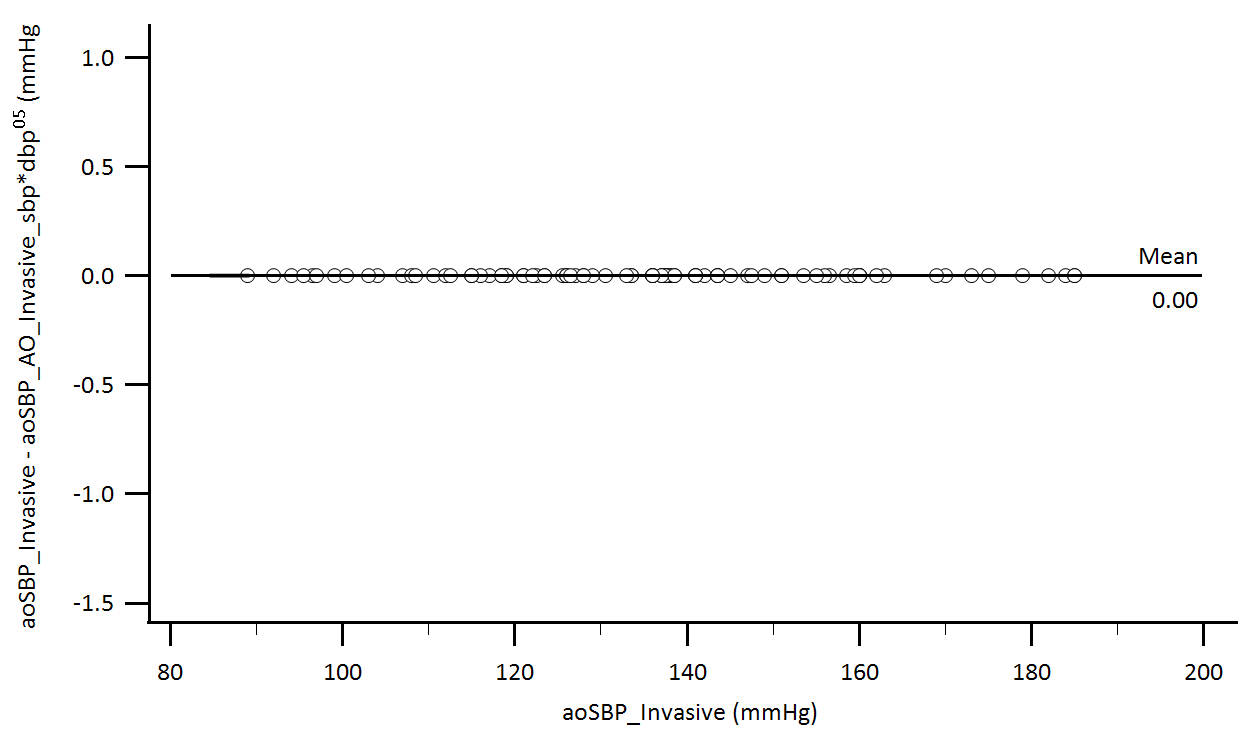


**Figure S2.** Agreement between aoSBP levels (i) measured invasively (catheterization) and (ii) calculated (aoSBP=bMBP^2^/bDBP) from invasive measurements of bSBP and bDBP: concordance correlation plots (Top) and Bland-Altman plots (Bottom)

Concordance correlation coefficient plots


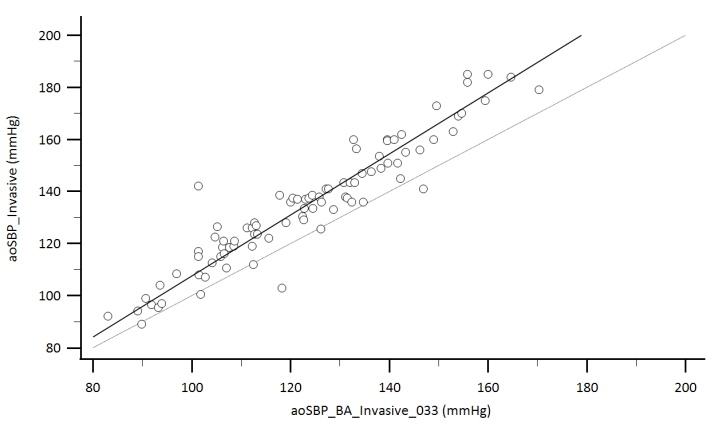

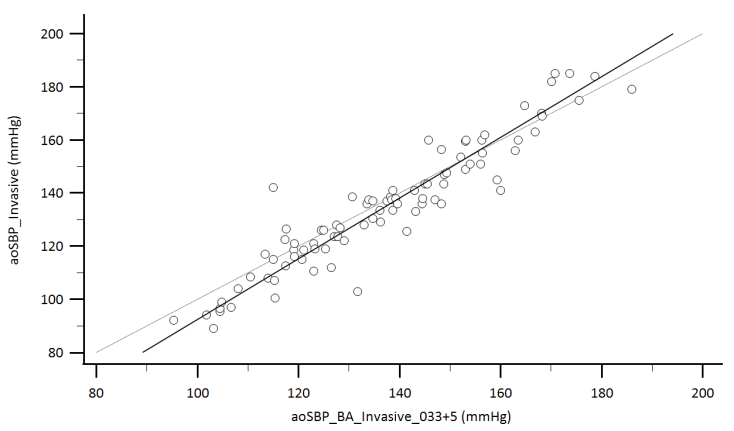


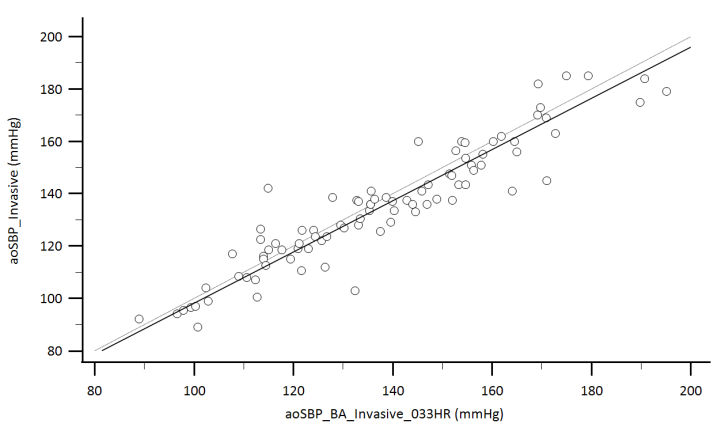

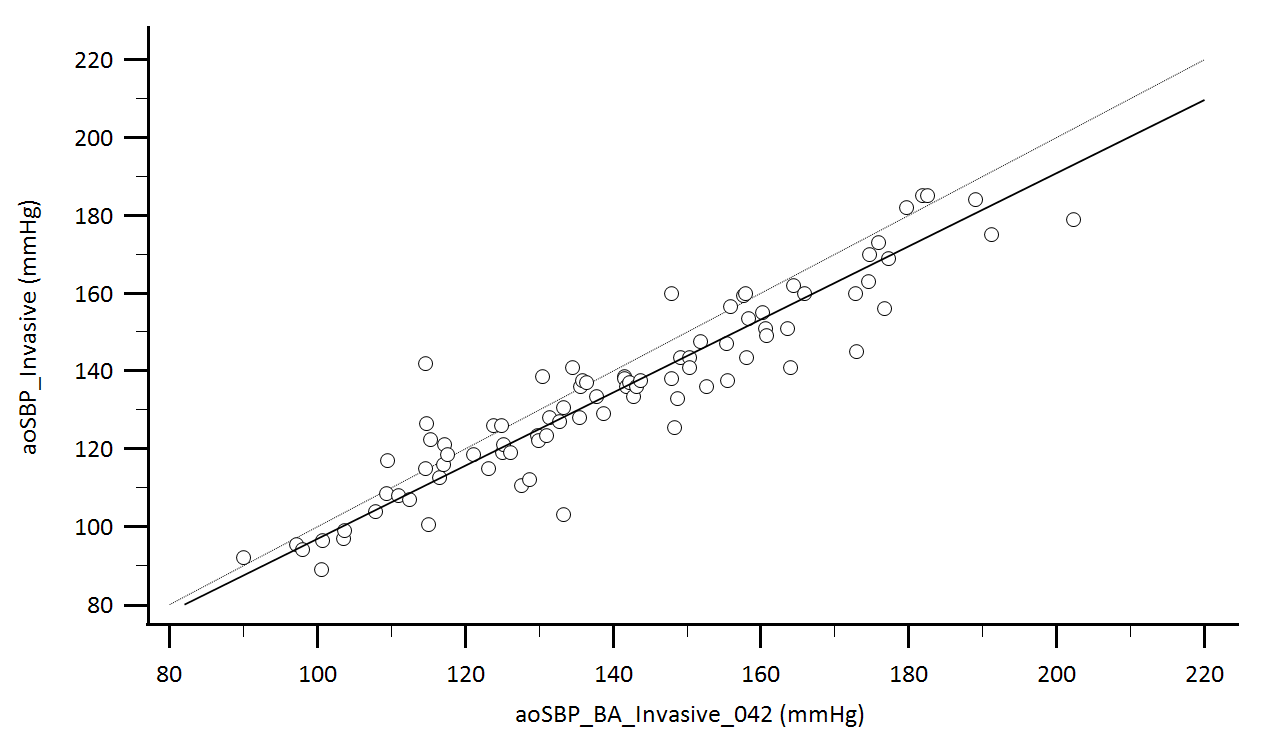


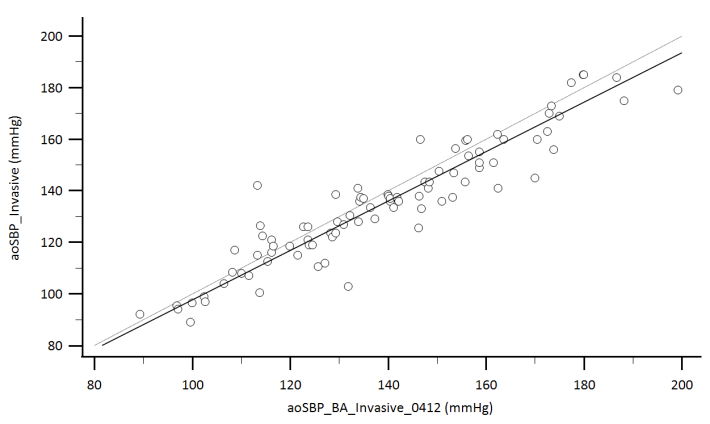

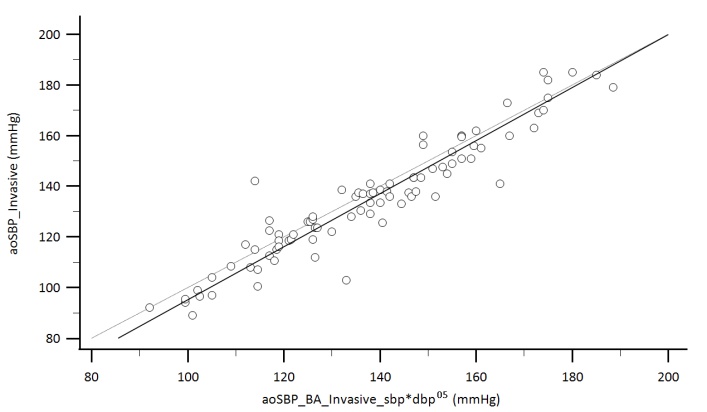


Bland Altman plots


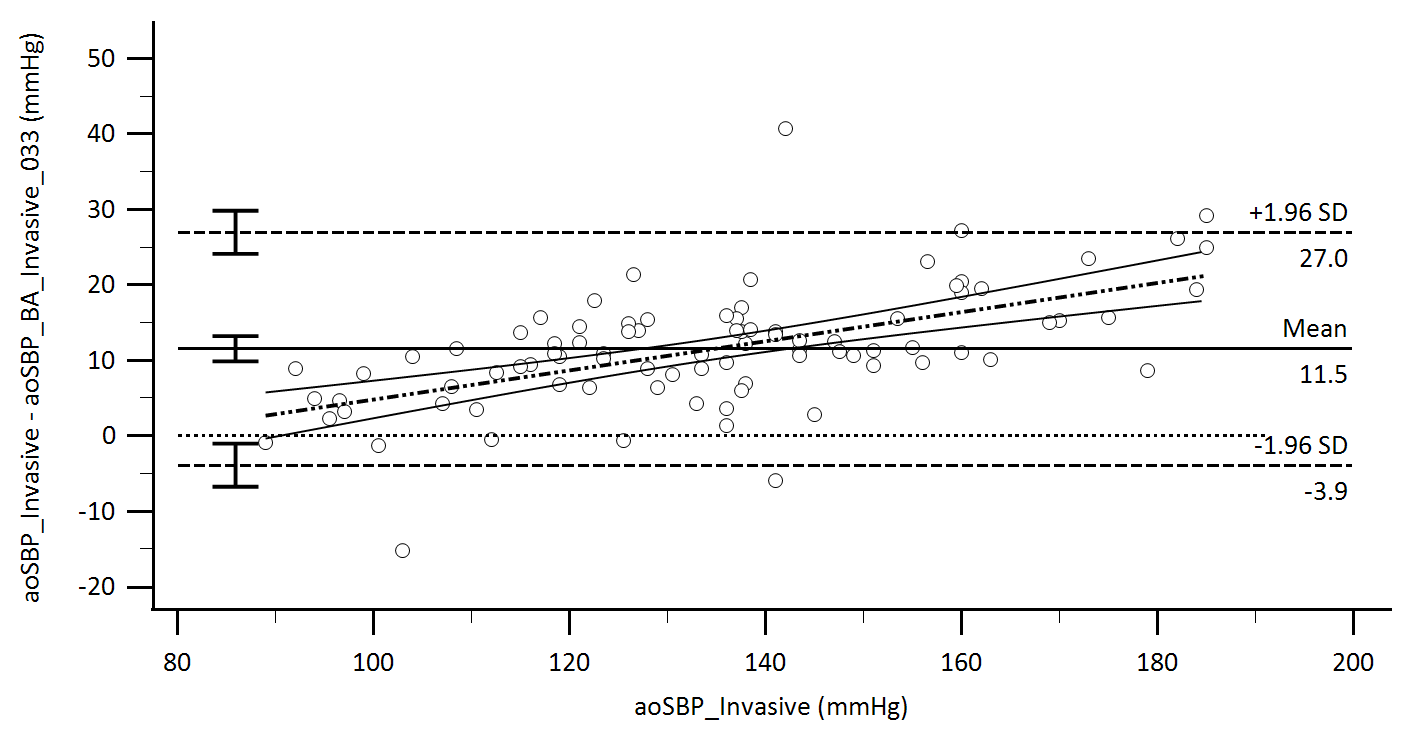

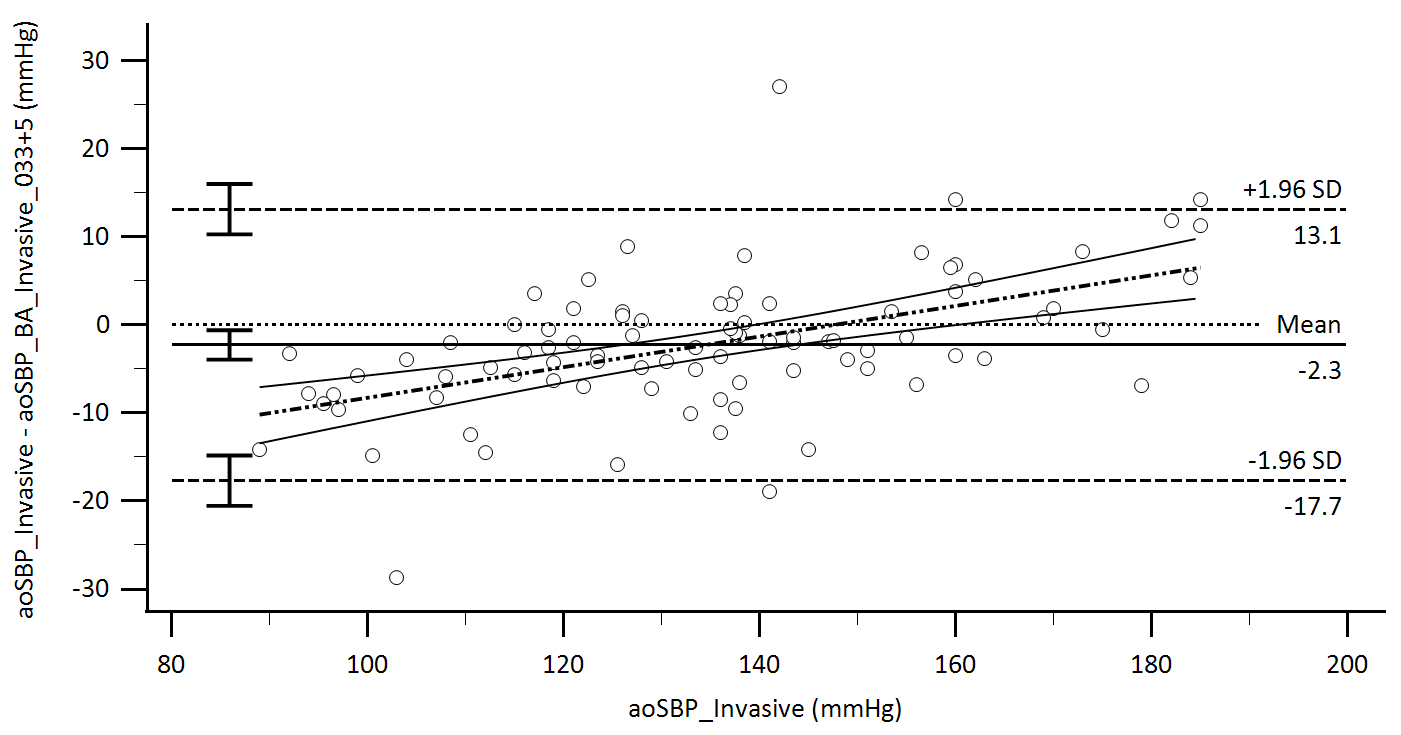


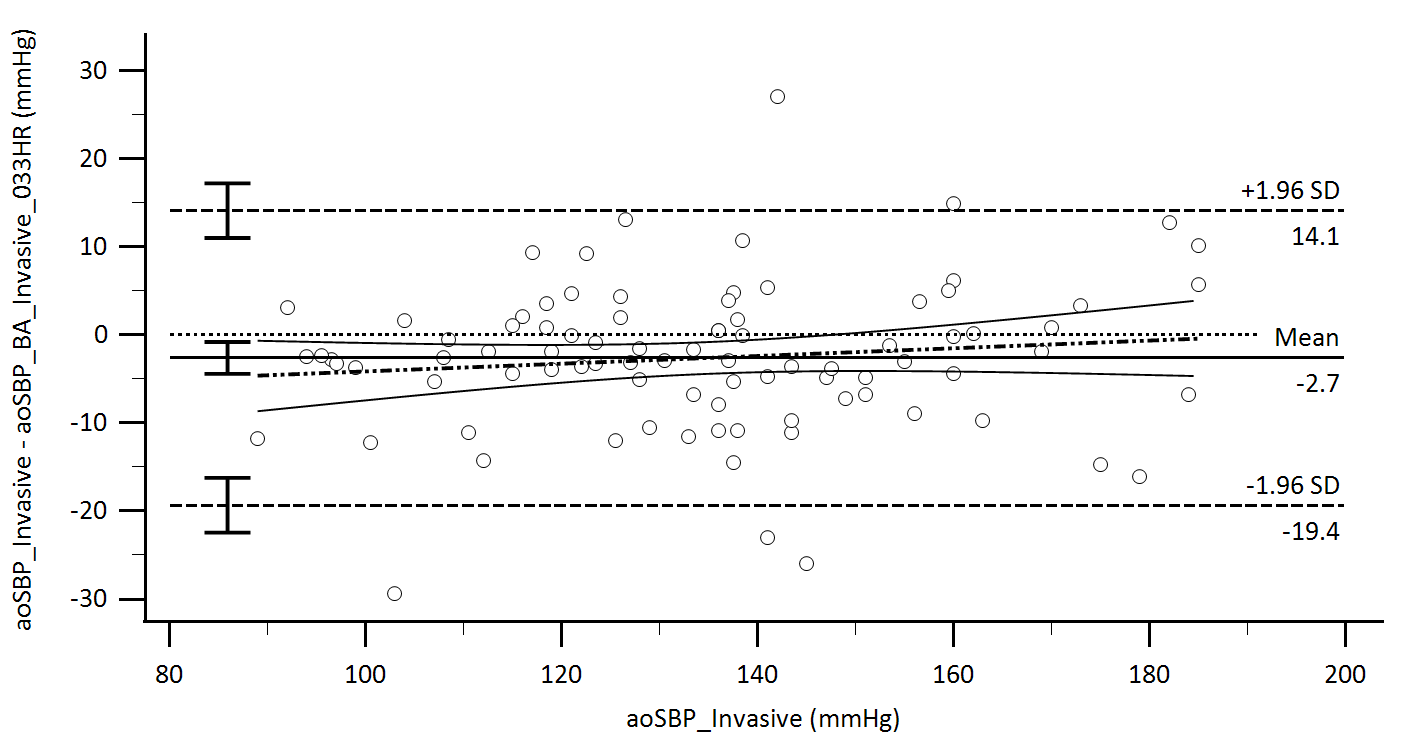

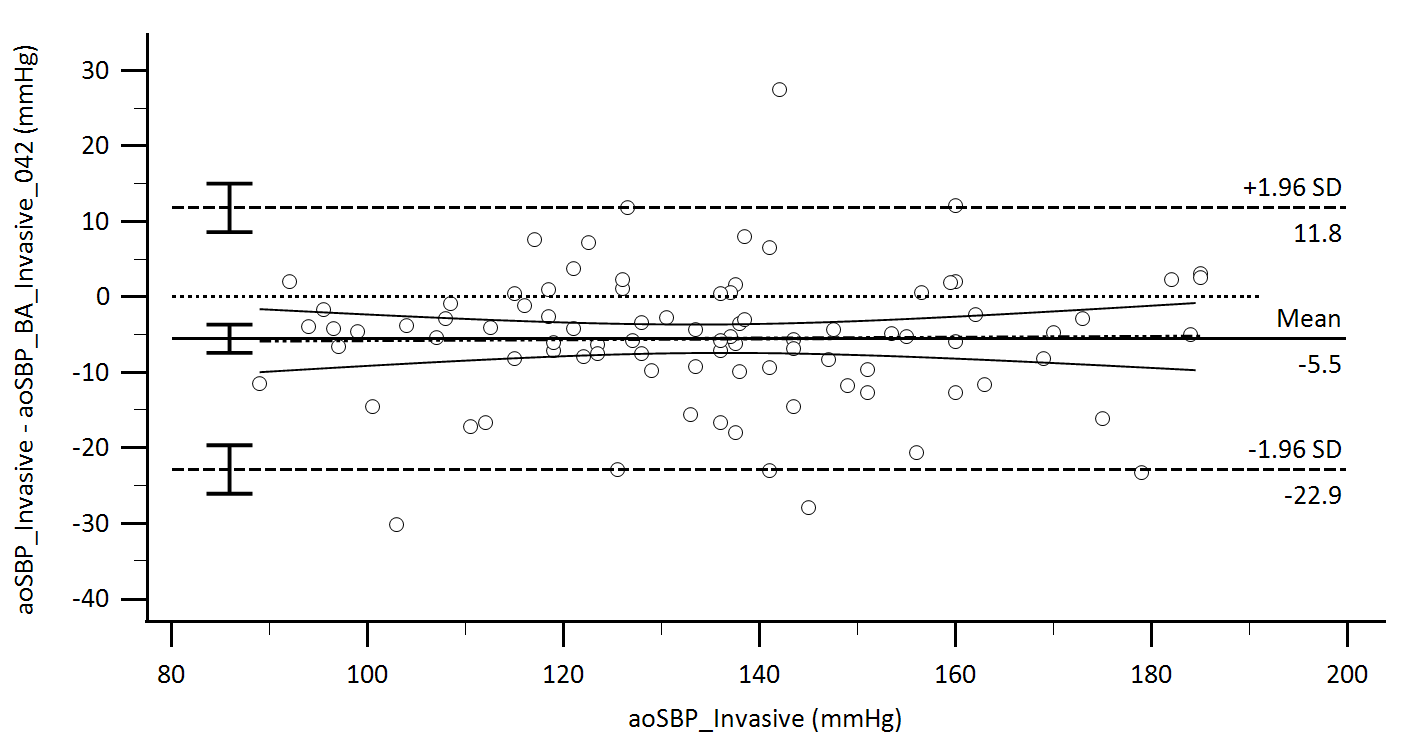


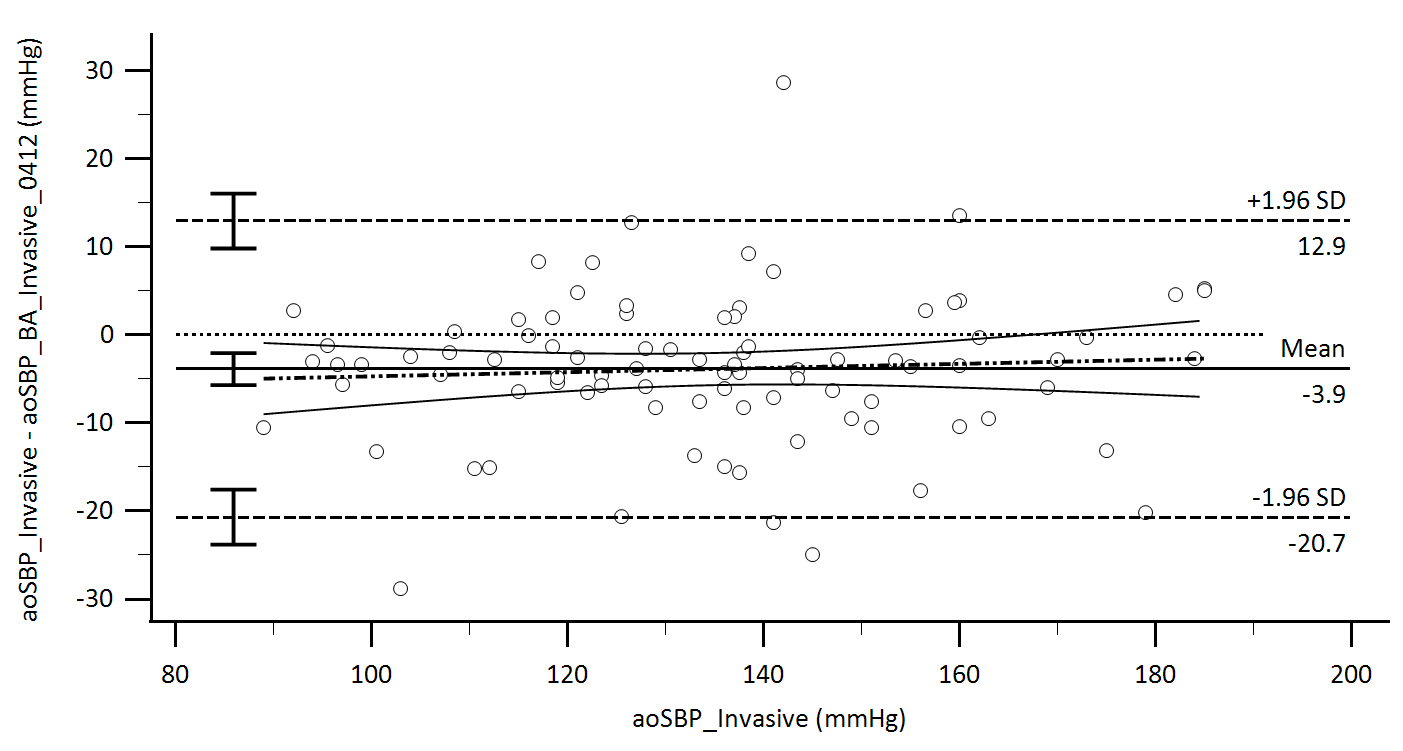

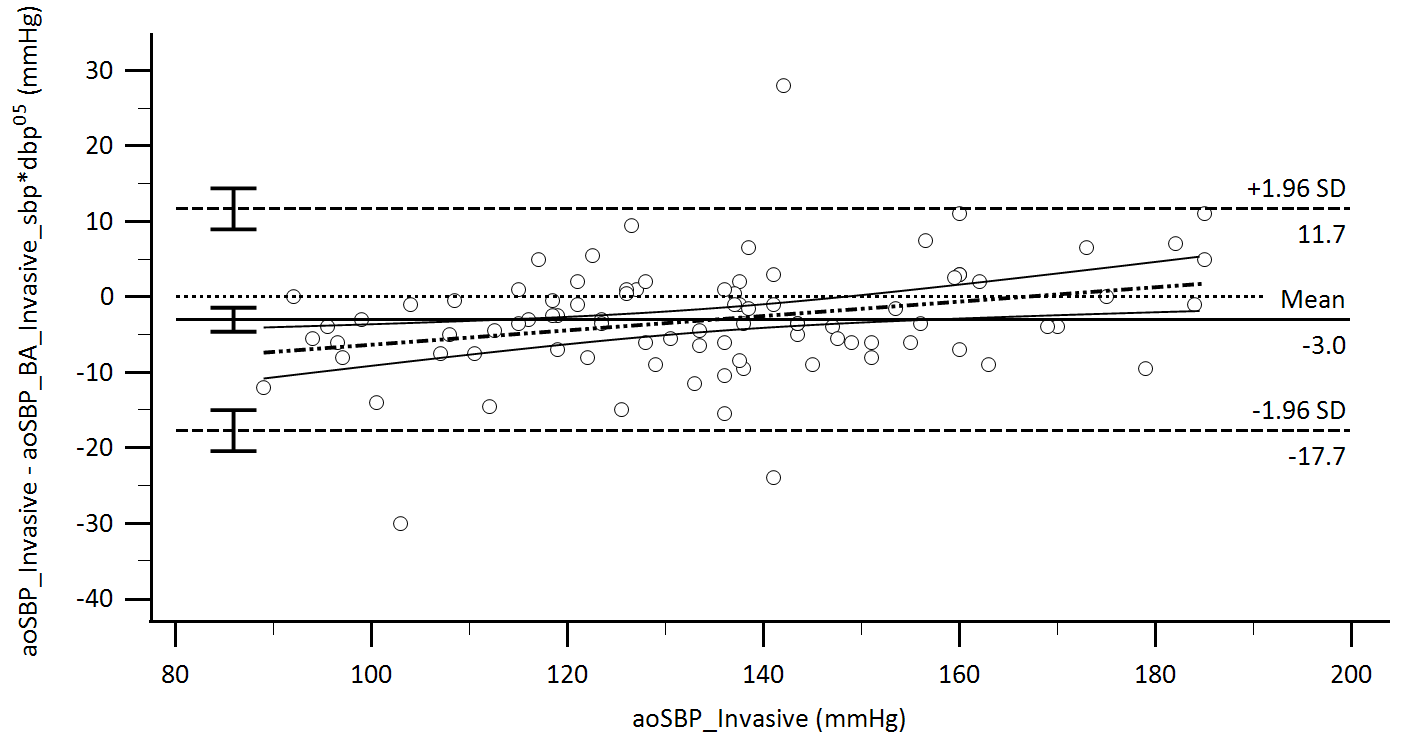


**Figure S3**. Agreement between aoSBP levels (i) measured invasively (catheterization) and (ii) calculated (aoSBP=bMBP^2^/bDBP) from non-invasive measurements of bSBP and bDBP: concordance correlation plots (Top) and Bland-Altman plots (Bottom)

Concordance correlation coefficient plots


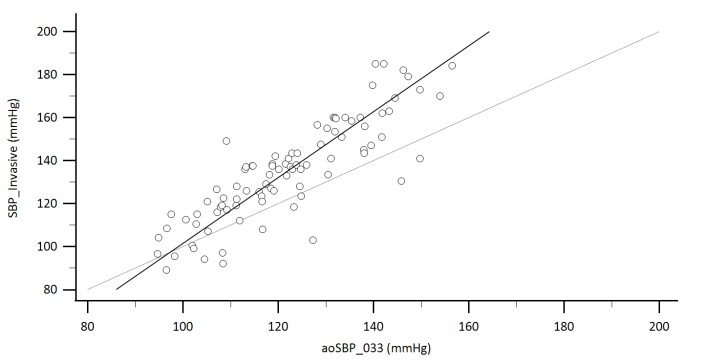

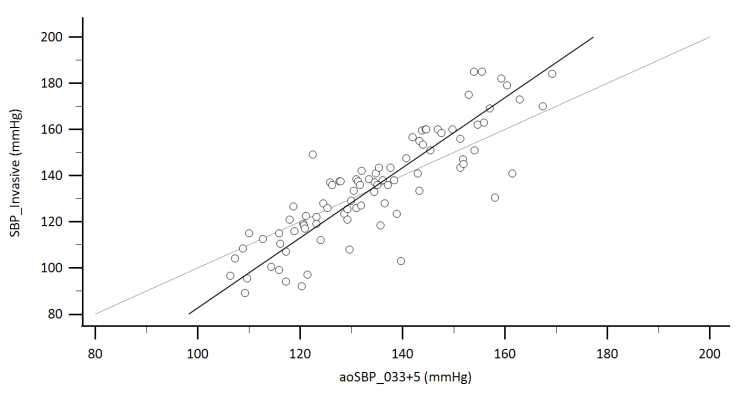


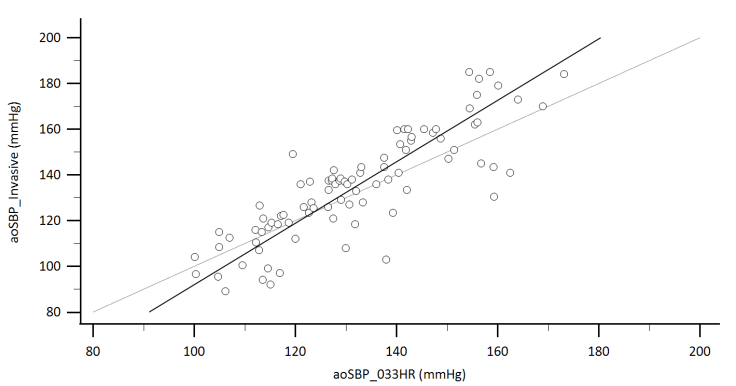

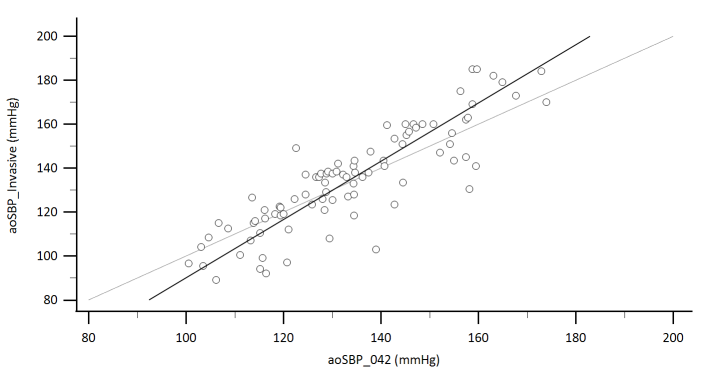


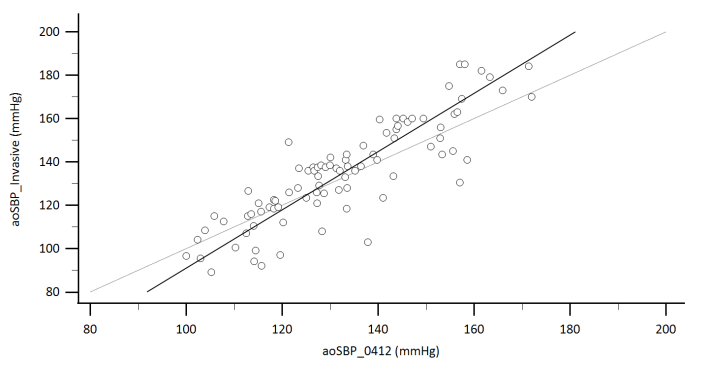

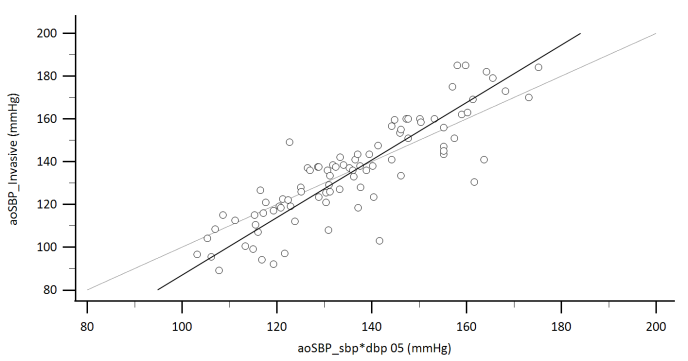


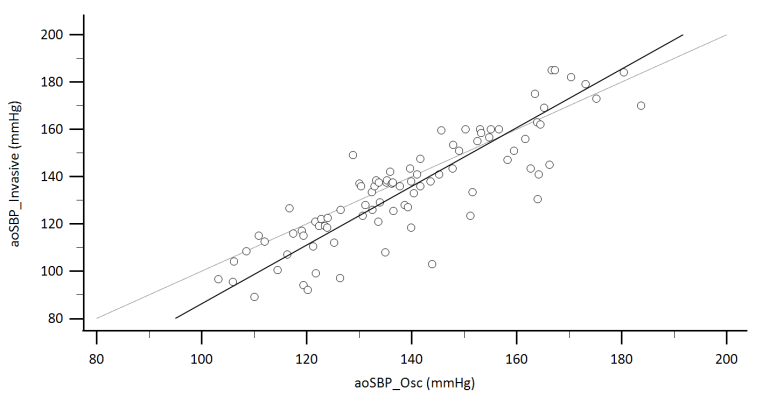


Bland Altman plots


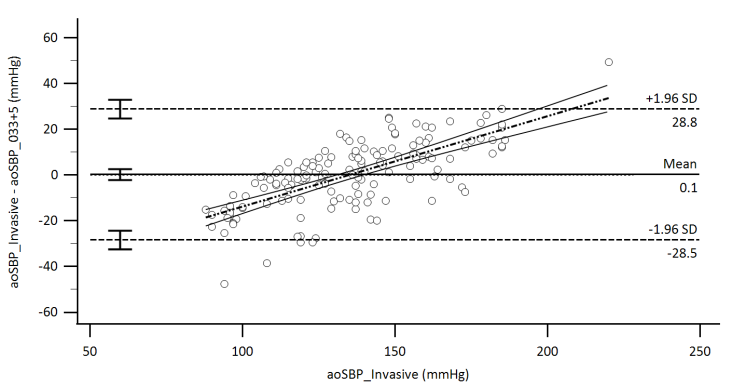

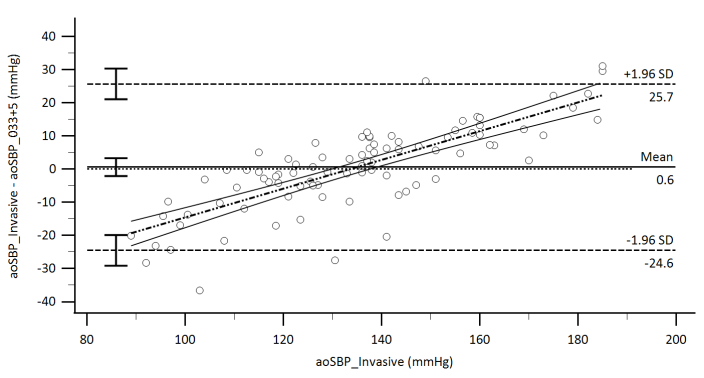


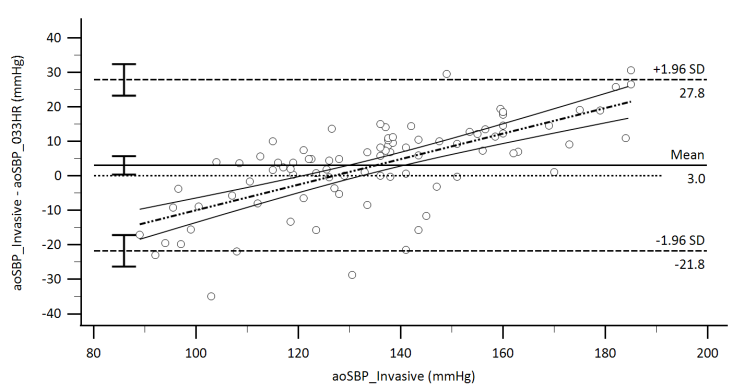

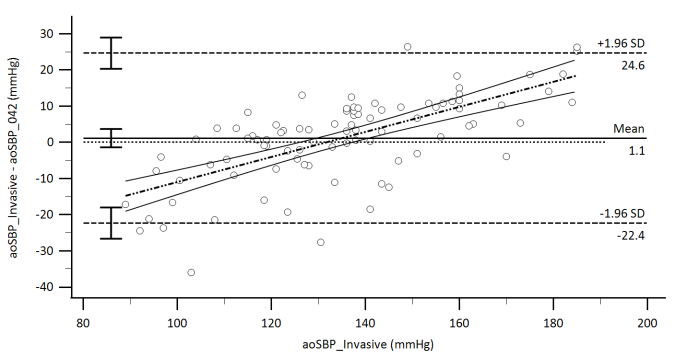


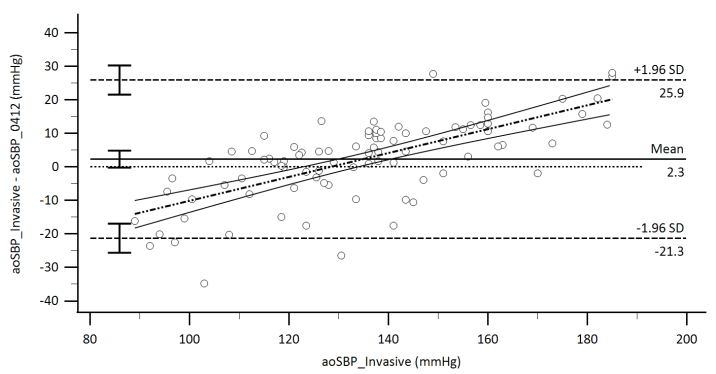

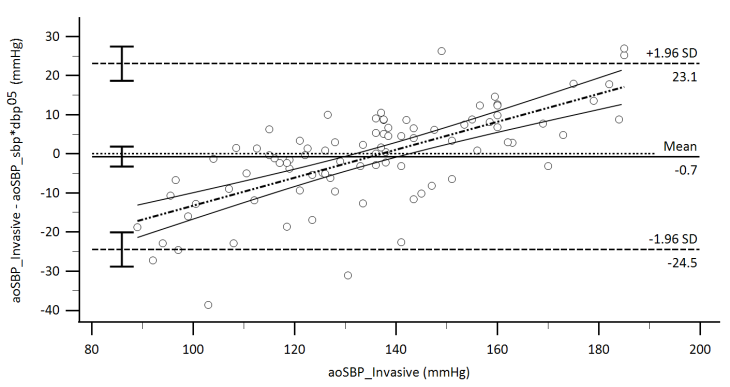


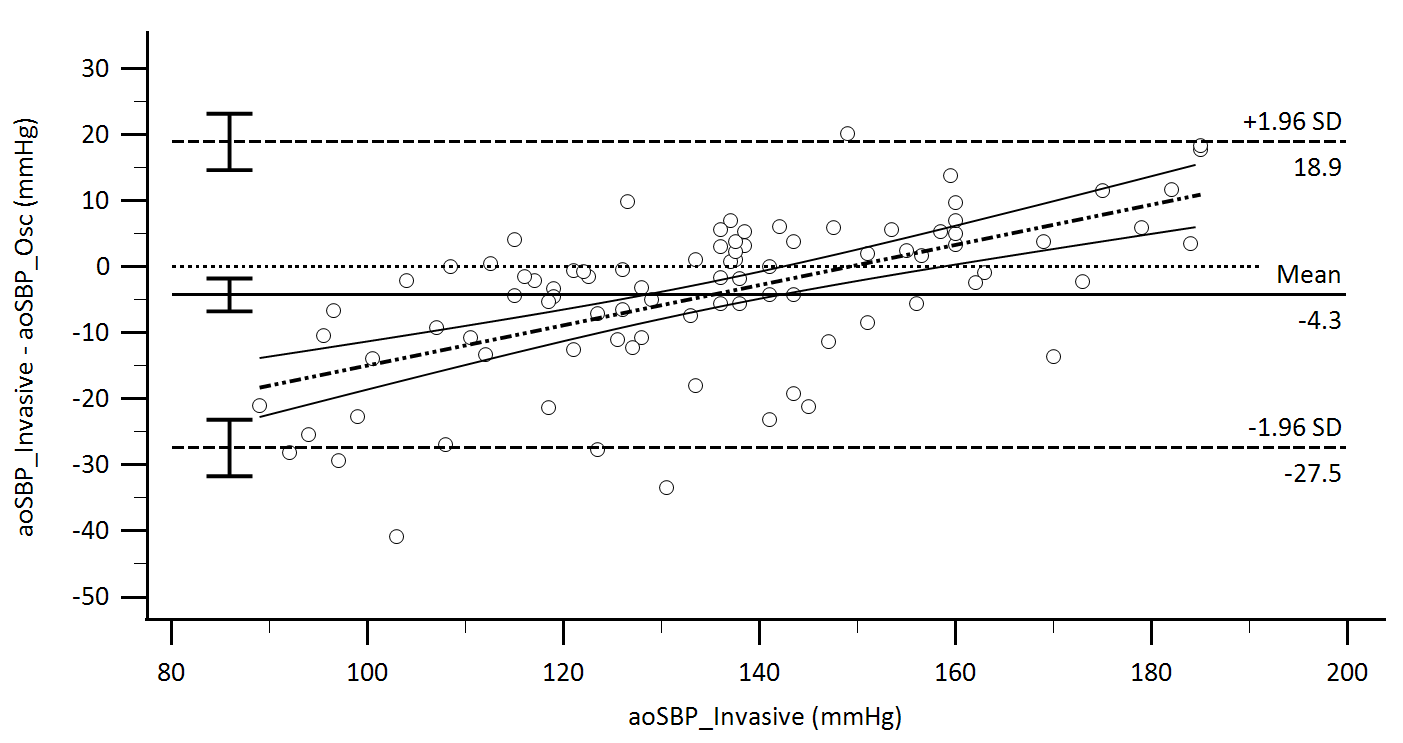

Supplement: Supplementary file 2 [file Table2.docx]
